# TM-Aligner: Multiple sequence alignment tool for transmembrane proteins with reduced time and improved accuracy

**DOI:** 10.1038/s41598-017-13083-y

**Published:** 2017-10-02

**Authors:** Basharat Bhat, Nazir A. Ganai, Syed Mudasir Andrabi, Riaz A. Shah, Ashutosh Singh

**Affiliations:** 1 Department of Life Science, Shiv Nadar University, Greater Noida, UP 201314 India; 2 Department of Animal Biotechnology, Sher-e-Kashmir University of Agricultural Sciences and Technology, Shuhama, Jammu and Kashmir 190016 India

## Abstract

Membrane proteins plays significant role in living cells. Transmembrane proteins are estimated to constitute approximately 30% of proteins at genomic scale. It has been a difficult task to develop specific alignment tools for transmembrane proteins due to limited number of experimentally validated protein structures. Alignment tools based on homology modeling provide fairly good result by recapitulating 70–80% residues in reference alignment provided all input sequences should have known template structures. However, homology modeling tools took substantial amount of time, thus aligning large numbers of sequences becomes computationally demanding. Here we present TM-Aligner, a new tool for transmembrane protein sequence alignment. TM-Aligner is based on Wu-Manber and dynamic string matching algorithm which has significantly improved its accuracy and speed of multiple sequence alignment. We compared TM-Aligner with prevailing other popular tools and performed benchmarking using three separate reference sets, BaliBASE3.0 reference set7 of alpha-helical transmembrane proteins, structure based alignment of transmembrane proteins from Pfam database and structure alignment from GPCRDB. Benchmarking against reference datasets indicated that TM-Aligner is more advanced method having least turnaround time with significant improvements over the most accurate methods such as PROMALS, MAFFT, TM-Coffee, Kalign, ClustalW, Muscle and PRALINE. TM-Aligner is freely available through http://lms.snu.edu.in/TM-Aligner/.

## Introduction

Transmembrane proteins or integral proteins are known for the variety of role they play inside the cellular system like communication, metabolism and regulation. Approximately 30% of proteins encoded by the mammalian genome are transmembrane proteins^1^. Interestingly, half of the drug molecules produce some effect on transmembrane proteins, another reason transmembrane proteins are so critical. Transmembrane proteins also participate in variety of cellular processes such as cell adhesion, immune-protection, metabolism and signal transduction^2^. Besides, transmembrane proteins are potential drug target candidates due to their essential roles as transporters, receptors and structural proteins as well as their effect on downstream intracellular processes^3^. Complex nature and involvement of transmembrane proteins in wide variety of biological processes makes them an imperative research subject. Transmembrane proteins are well known for their complexities in determining their structures experimentally^4^. Only 3099 transmembrane protein structures are available till date with Protein Data Bank of transmembrane proteins version 2017.02.10 ^5^. This lack of data inspired many research groups towards predicting structures of transmembrane proteins by homology modeling. In homology modeling, unknown structure of a target sequence is modeled on a known (template) structure of a distantly-related protein, in order to gain insights into membrane protein function. Such studies rely on methods for detecting relationships between two proteins, by subsequently, aligning their protein sequences. Moreover, wide variations can be detected at the sequence level within a transmembrane protein family, thereby increasing complexity and error in the alignment.

Multiple sequence alignment of transmembrane proteins was first addressed by Cserzo^6^ followed by Bahr^7^, and over the years, a few more methods and tools were developed for transmembrane protein sequence alignment. Multiple sequence alignment (MSA) methods, like Kalign^8^, MAFFT^9^, Muscle^10^, and ClustalW derives their accuracy from a ‘consistency’ criterion and/or iterative optimization. Consistency-based approaches aim to generate a multiple sequence alignment that accords best with a library of pairwise alignments between the sequences being aligned. TM-Coffee^11^, PRALINETM^12^ and Promals^13^ are based on homology modelling^14^ that has been found to perform well on alignments of transmembrane proteins from the BALiBASE2.0^7^ benchmark. Dearth of known transmembrane proteins structures in PDB often leads to low sequence identity in best templates, which is often under 30%. Despite availability of homology based tools for multiple sequence alignment of transmembrane proteins, it is likely that a significant number of transmembrane regions remain undetected or unaligned because of limitations of the available methods like number of input sequences, turnaround time and dependency on structures. On the other hand, TM-Aligner is not working on structural homology based approaches neither it has limitation over number of sequences and took very less turnaround time. TM-Aligner can perform multiple sequence alignment of unlimited number of transmembrane proteins of any length.

As biological membrane proteins have a transmembrane between cytoplasmic and non-cytoplasmic regions, so even at low sequence similarity, accurate alignment is possible by dividing the sequence into different regions and aligning them separately. These alignments are then stitched together precisely so that transmembrane regions were not disrupted and important residues within protein family are conserved throughout the alignment process. TM-Aligner is an unconditional (in terms of length and number of sequences) tool which can align transmembrane proteins accurately and responsively. TM-Aligner has been designed as a unique global, progressive alignment method for aligning transmembrane proteins. Progressive or tree-based method align most similar sequences first and then successively add less similar sequences to alignment until all sequences are aligned. TM-Aligner uses UPGMA^15^ method to create an initial guide tree that describes sequence relatedness. To predict transmembrane regions, TMHMM^16^ was used and alignments were made using dynamic programming and Wu-Manber string matching algorithm^17^ to stitch different regions together.

## Method

### TM-Aligner implementation

**Figure 1:**
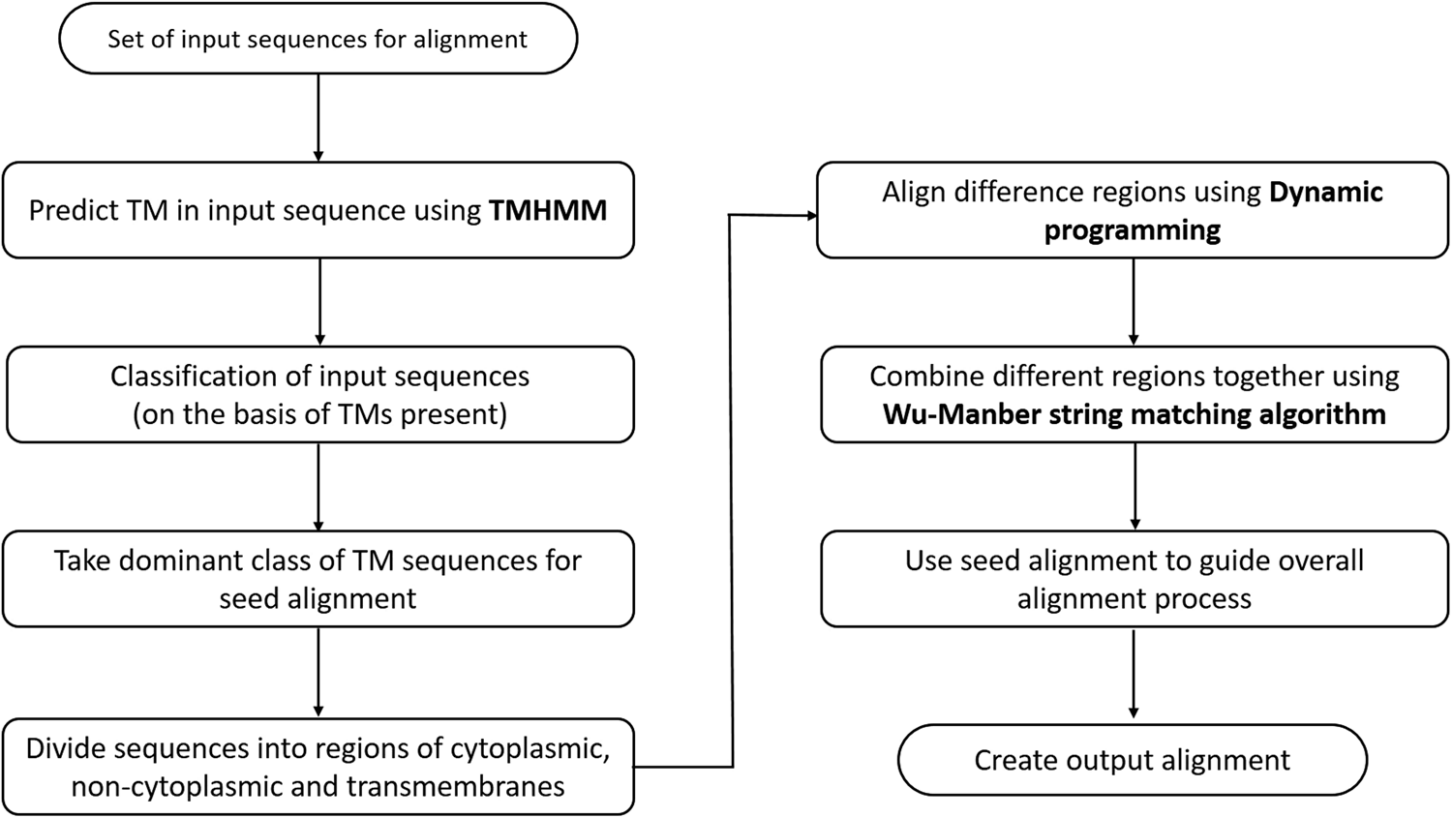
TM-Aligner workflow on a set of input sequences. Here TM-Aligner predicts transmembrane, cytoplasmic and non-cytoplasmic regions from input sequences using TMHMM, input sequences are then classified into different groups based on the number of TMs present in each sequence. Classes with the dominant number of transmembrane sequences were chosen for alignment which were then used as a seed alignment for overall alignment process.

TM-Aligner (Transmembrane Membrane proteins - Aligner) is a protein sequence alignment tool developed in C, Perl (version 5.20) and PHP (version 5.6). The web interface of TM-Aligner is written in PHP and JavaScript under XAMPP web server running on a Linux system. TM-Aligner uses the progressive alignment strategy for aligning protein sequences. The UPGMA method is used to find similar sequences which guide the alignment process. Time complexity of UPGMA is O(N^3^), however, time complexity has been reduced to O(N^2^) by maintaining an array of references to the minimum value in each row of the distance matrix^10^. TMHMM is used to predict transmembrane regions within the protein sequence. The input protein sequences are divided into cytoplasmic, non-cytoplasmic and transmembrane regions. For aligning divergent sequences, dynamic programming has been found exceptionally superior over K-tuple method therefore, all regions are aligned independently using dynamic programming. The Wu-Manber string matching algorithm is used in stitching transmembrane regions with cytoplasmic and non-cytoplasmic regions. Wu-Manber string matching algorithm sieve through thousands of matches that are found in sequences (or profiles) and determine the largest set of consistent matches that can be included in final alignment. The workflow for alignment process is outlined in Fig. 1.

### Dynamic programming

Dynamic programming^18^ is most stringent and demanding in terms of memory usage and CPU time. To reduce the time taken by dynamic programming, an additional matrix of size (m + 1) * (n + 1) (‘m’ and ‘n’ is the size of sequences to be aligned) has been introduced, called branch matrix which stores transitions occurring in every cell of dynamic programming matrix. Therefore, optimal alignment is obtained from branch matrix. Since TM-Aligner breaks input sequence into short sequences, memory optimization is not required. All these steps reduce the processing time in Dynamic programming.

### Wu-Manber algorithm

Wu-Manber is a high performance^8,17,19^ multi-pattern matching algorithm, which uses text in blocks of size S (usually 2 or 3) for comparison. Wu-Manber algorithm has two core mechanisms, filtering based on hashing and blocking based on bad—shift mechanism.

Wu-Manber works in two phases, preprocessing phase and scanning phase.

### Preprocessing Stage

Preprocessing phase speed up process of pattern matching, by determining the size of match window which is equal to the smallest length pattern (say ‘m’) and creating three important tables, SHIFT table, HASH table and PREFIX table. Wu-Manber algorithm uses patterns of a size S to create a SHIFT table, when SHIFT is 0. HASH and PREFIX tables are used to identify candidate pattern.

### Scanning Stage

Pattern search works as:

1. Locating match window at the start of the sequence.
2. Compare last S characters of the window against character blocks in SHIFT table. If corresponding value in SHIFT table is greater than zero than window is shifted according to value and process is repeated. Otherwise, HASH table is used for a match within matching window.
3. If HASH table consists multiple entries than match prefix of a pattern from prefix table, if it is matched, complete pattern were matched.
4. Continue the process till end of the text.

### Scoring

In TM-Aligner transmembrane, cytoplasmic and non-cytoplasmic regions are predicted and aligned using dynamic programming. All regions are aligned independently. 3 substitution matrix (PHAT, BLOSUM62 and GONNET250) are provided for multiple sequence alignment, default is PHAT with gap insertion penalty of 8 and gap extension penalty of 1.

## Results

### Benchmarking

To compare TM- Aligner to other alignment programs, eight transmembrane protein families of BAliBASE3.0 reference set7 (which is a gold standard for multiple sequence alignment benchmarking), multiple datasets from Pfam database (Version 31, release date March, 2017)^20^ and structure based alignment from GPCRDB (release date July 25, 2017)^21^ has been used.

#### BALIBASE3.0

BAliBASE^22^ test sets are a collection of alignments derived from structural databases and/or manual alignment from literature. In BAliBASE, alignment of transmembrane proteins was constructed from alignment of known proteins families and new sequences were added, based on score obtained in profile search^7^. References set 7 of BAliBASE version- 3.0 has been implemented for benchmarking which contains 435 alpha-helical transmembrane proteins, classified into eight super-families, namely 7tm, acr, photo, dtd, ion, msl, Nat and ptga, each multiply aligned. The accuracy of the method was assessed by sum of pairs score (SP), which reflects the percentage of correctly aligned residues with respect to reference alignment. Total Column score (TC) were not considered for scoring purpose because this score did not reflect the biological correctness of alignments. For example, consider a sequence alignment where the most of the sequences were correctly aligned, the total column score can end up noticeably zero because of a single misaligned sequence^8^.

#### Pfam Database

Pfam^20^ is a database of conserved protein families, containing collection of multiple sequence alignment and profile hidden markov models. In Pfam, seed alignment was constructed from representative protein sequences of family, to accurately identify the position-specific amino acid frequency, gap penalty and length parameter in profile hidden markov model. Other sequences were added on the basic of profile alignment score. For TM-Aligner, alignments from multiple TM families containing 9735 distant sequences were used for benchmarking.

### Comparative Analysis

TM-Aligner is very quick and exclusively well suited for aligning large numbers of sequences.TM-Aligner was compared with seven most accurate alignment methods: i. PRALINETM one of the most widely used alignment tool for aligning transmembrane proteins; ii. TM-Coffee, which has the best average SP score on BAliBASE, reported till date; iii. Promals uses progressive alignment strategy for MSA of protein sequences by incorporating profile information from known structure databases and secondary structure prediction methods, iv. Muscle, v. ClustalW, vi. MAFFT and vii. Kalign. These all are based on dynamic programming method, progressive alignment and iterative refinement (all methods are tested with default parameters i.e. without changing substitution matrix gap opening penalty and gap extension penalty). For TM- Aligner benchmarking BAliBASE3.0 reference set-7 has been used, which is the only reference set for transmembrane proteins in BAliBASE. For comparison, Sum-of -Pair (SP) score and processing time were considered for each family in BAliBASE3.0 reference set – 7 (Table 1). P-value were calculated using paired t-test. The SP score of TM-Aligner was also found better, than the tools that were developed using BAliBASE i.e. Muscle by 2.6% (p-value = 0.039668335) and ClustalW by 8.6% (p-value = 0.039668335).

**Table 1:** Performance comparison between TM-Aligner and other MSA tools on each BAliBASE3-reference set7 protein family: a) Sum-of-Pair (SP) score b) Time - indicate processing time/CPU time in seconds. Standalone version of PRALINETM is unavailable, so praline is not included in time comparison table; however, the time taken by PRALINETM is greater than TM-Coffee. Every other tool including TM-Aligner is tested individually using single threaded machine with two available cores.

(a) *SP SCORE*		***Alignment tools***							
*Family*	*No. of Seq*.	*Praline TM*	*TM-Coffee*	*PROMALS*	*ClustalW*	*Muscle*	*Mafft*	*Kalign*	*TM-Aligner*
*PTGA*	51	0.652	0.738	**0.740**	0.461	0.519	0.630	0.321	0.700
*ACR*	43	0.914	0.946	0.910	0.906	**0.950**	0.914	0.916	0.919
*MSL*	14	0.838	0.839	0.847	0.864	0.865	0.829	0.704	**0.888**
*DTD*	55	0.859	**0.880**	0.850	0.786	0.869	0.829	0.501	0.870
*PHOTO*	33	0.897	0.911	0.905	0.887	0.901	0.857	0.501	**0.916**
*ION*	52	0.319	**0.540**	0.500	0.354	0.514	0.538	0.285	0.509
*NAT*	59	0.773	0.718	0.747	0.630	0.741	0.644	0.275	**0.754**
*7TM*	128	0.813	**0.884**	0.832	0.847	0.847	0.806	0.480	0.815
*AVERAGE*		0.758	0.807	0.790	0.710	0.770	0.755	0.490	0.796
**(b)** ***TIME (in seconds)***		**Alignment tools**							
***Family***	***No. of Seq*** *.*		***TM-Coffee***	***PROMALS***	***ClustalW***	***Muscle***	***Mafft***	***Kalign***	***TM-Aligner***
*PTGA*	51		778	17633	5	28	38	3	17
*ACR*	43		1836	35622	8	28	35	6	26
*MSL*	14		17	1055	1	3	12	1	3
*DTD*	55		1443	21885	6	32	44	3	24
*PHOTO*	33		38	3962	1	3	26	1	7
*ION*	52		1385	18521	4	78	45	6	26
*NAT*	59		602	21055	6	32	54	3	21
*7TM*	128		4346	35865	19	52	117	6	56
*AVERAGE*			1300	19500	6	32	46	3	22

TM-Aligner outperforms Praline by 3.8% on the basis of SP- score. TM-Aligner and Promals have similar accuracy, however, Promals is computationally very demanding. On average Promals takes several thousand fold more CPU time than TM-Aligner (p-value = 0.00115), Table 1b. TM-Coffee outperforms TM-Aligner by 1.1% for sum -of-pair score. However, the significance of the improvement is not very strong (P-value = 0.469498). TM-Coffee being the most responsive homology modelling based tool in aligning transmembrane sequences takes 60% more CPU time than TM-Aligner (P-value = 0.017452). Our study has established that TM-Aligner is a much more efficient tool in terms of accuracy, speed and number of input sequences when aligning large amounts of transmembrane sequences or distant sequences.

#### Large Dataset

As BAliBASE alignments are relatively small, large alignments from Pfam database has been used for examining the performance of TM-Aligner. For that, multiple test sets from Pfam database were used. Here, the comparative analysis is limited to tools which works on the basis of homology modeling. The result in Table 2 strongly supports result in Table 1 and clearly shows TM-Aligner is as accurate as homology based transmembrane alignment tools. Surprisingly, homology based alignment tools could not complete all alignments for large datasets.

**Table 2:** Performance comparison (in terms of SP-Score) between TM-Aligner and other transmembrane alignment tools on Pfam alignments. ‘x’ - represents, alignment could not be completed either due to restriction on number of input sequences or resource limitation.

Pfam ID.	Number of Seq.	TM-Aligner	TM-Coffee	Praline	Promals
PF01036)	1038	**0.721**	x	x	0.708
PF10316	434	**0.909**	x	0.658	0.708
PF14778	424	**0.822**	x	0.706	0.759
PF01534	1894	**0.900**	x	x	x
PF02117	182	0.812	**0.840**	0.711	0.810
PF10325	372	0.737	x	0.608	0.100
PF10413	177	**1.000**	**1.000**	**1.000**	**1.000**
PF02076	981	**0.820**	x	x	0.557
PF02714	3894	0.510	x	x	x
PF02116	261	0.900	0.910	0.892	**0.920**
PF03383	78	0.540	**0.550**	0.485	0.517

Another benchmarking approach has been used against structural based alignment from GPCRDB (which collect, combine and validate data on G protein coupled receptors) for evaluating performance of TM-Aligner details and result is provided in Table 3.

**Table 3:** Performance comparison between TM-Aligner and other transmembrane alignment tools on GPCRDB structural alignments.

Family	No. of sequences	TM-Aligner	Praline	TM-Coffee	Promals
Human GPCR protein sequences	398	0.430	0.261	0.284	0.201
ClassA GPCR protein sequences*	194	0.841	0.797	0.839	0.802

*Only TM regions were used for benchmarking.

Detailed comparison of TM-Aligner with the available transmembrane alignment tools is shown in Table 4.

**Table 4:** TM-Aligner compared with other available transmembrane alignment tools.

ALIGNMENT TOOL	ALGORITHM USED	INPUT LIMITATION
TM-ALIGNER	TM-Prediction and Dynamic Alignment	**5000 (TO LIMIT SERVER LOAD)**
TM-COFFEE^10^	Homology modelling	**1000 SEQUENCE**
PRALINE^11^	Homology modelling	**500 SEQUENCES**
PROMALS^12^	Homology modelling	**NOT KNOWN**

## Discussion and Conclusions

In this work, we have shown how 2D structure prediction and string matching algorithms can increase alignment quality for transmembrane proteins. Our results (in Table 1, 2 and 3) suggests that TM-Aligner has accuracy similar to the tools based on homology-modeling, however, TM-aligner is superior to other transmembrane alignment tools in terms of computation time. Almost all the transmembrane protein alignment tools depend on template structures for alignment accuracy however, TM-Aligner is robust in aligning transmembrane sequences without any dependency over template structures. TM-Aligner when compared with other popular tools used for transmembrane protein sequence alignment, the average accuracy was found to be similar (Tables 1, 2 and 3) with that of TM-Aligner but, for large datasets, none of them were able to complete the alignment. TM-Aligner provides accurate results with least turnaround time which can be very useful for better classification of anonymous TM protein sequences and in identification of important residues within TM region.

Tables 1, 2 and 3 strongly suggests 2D structure prediction and dynamic programming can increase alignment quality for transmembrane proteins and can be implemented on bigger datasets with diverse sequences. TM-Aligner may help in classification of anonymous TM protein sequences and in identification of important residues within TM region.

## TM-Aligner Web server

Web server for TM-Aligner is simple and interactive; TM-Aligner accepts input in FASTA format. The user can directly paste protein sequence in the text-area provided or upload sequence file in FASTA format. The proposed maximum number of sequences that should be submitted to the server is set to 5000, but this is mainly to limit the server load and is not a program limitation.

**Figure 2:**
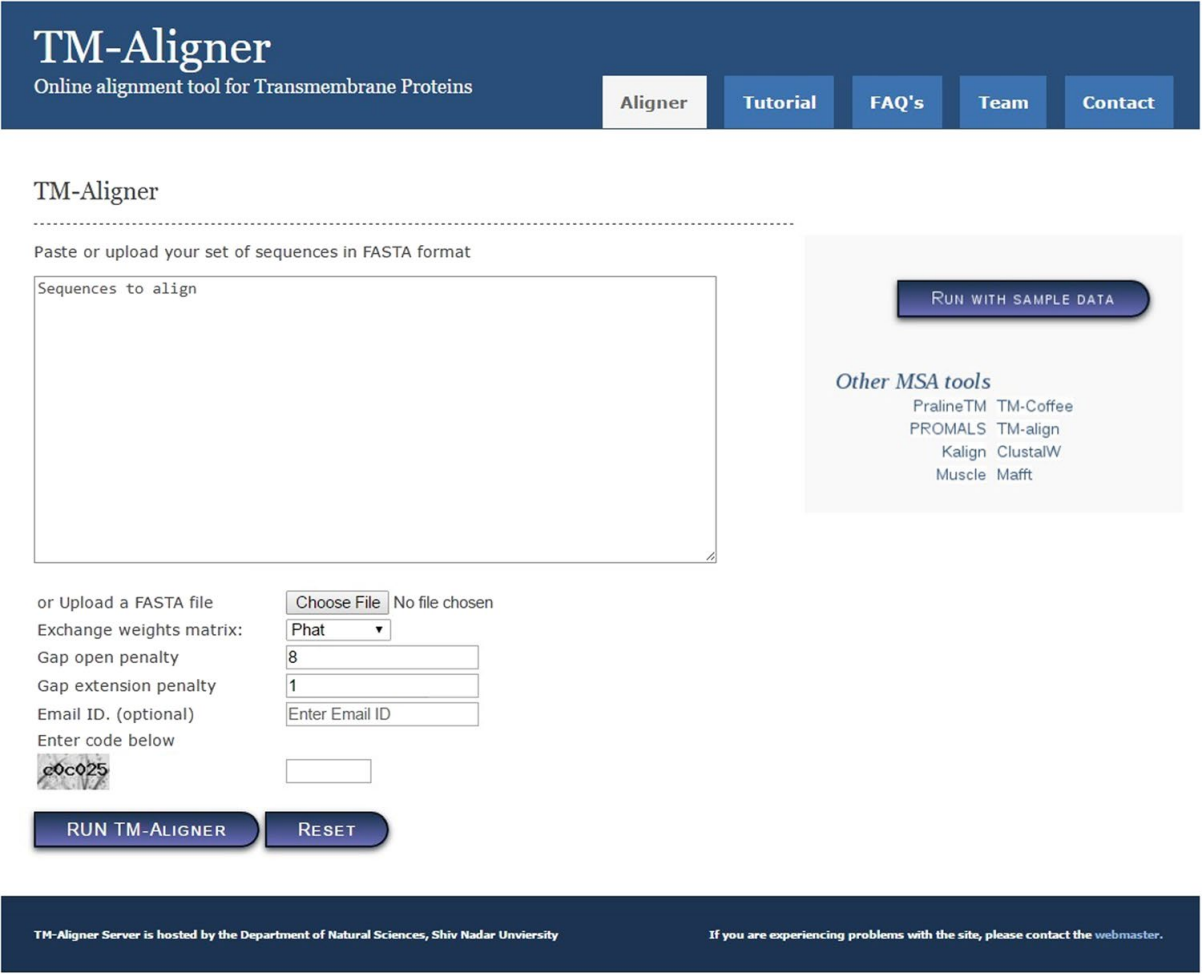
Front page of the TM-Aligner server. The main section allows the user to paste or upload sequences in fasta format. Options to modify alignment parameters, like substitution matrix, gap open and gap extension penalty are provided. A brief description of each option is available in the tutorial section inside navigation panel of web-server.

**Figure 3:**
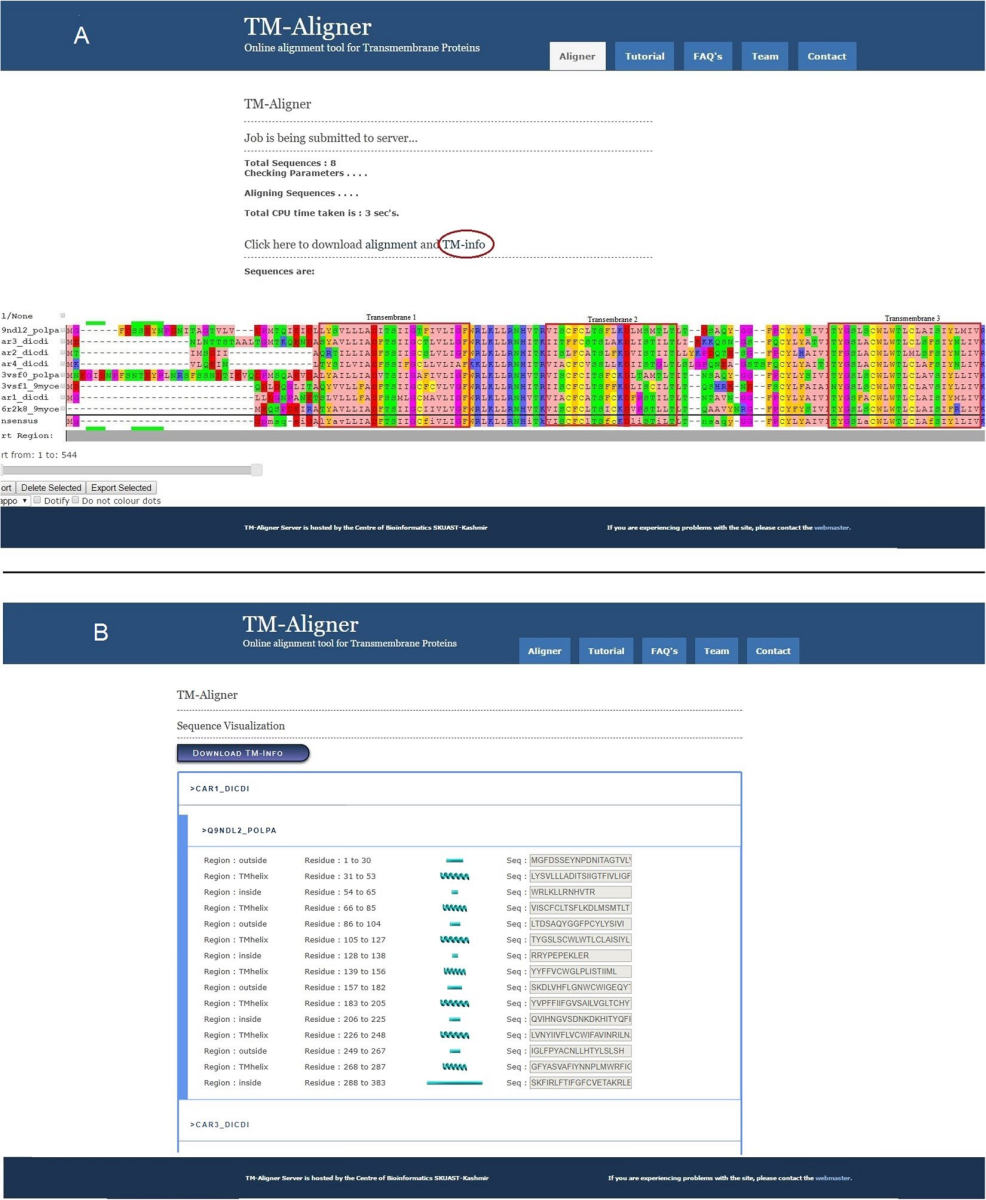
Colored alignment produced by TM-Aligner server. Input sequences are of cAMP receptor proteins. (**A)** Shows result page, TM-Aligner provides visualization of multiple sequence alignment in different color schemes and with a variety of options. “**TM-Info**” tab on the result page provides complete information about a total number of transmembrane present in the input sequences (**B**).

TM-Aligner is fast and robust alignment tool and provides instant result for alignment. An optional email notification can be requested that is delivered upon the completion of job and has the link to the results. Gap opening and gap extension penalties and the amino acid substitution matrix can be manually set if required (default is 8, 1 with PHAT matrix) for any of the alignment strategies as given in Fig. 2. The results page is automatically displayed, once the job is complete. TM-Aligner provides visualization of MSA in different color schemes and with variety of options. TM-Aligner provides an options to select and delete sequence(s) from final alignment; a consensus sequence provided at the bottom of alignment which gets updated automatically when alignment is changed (Fig. 3). All these options reduce the dependency of the user to use other software for alignment visualizing. TM-Info tab on the result page provides complete information about transmembranes present in the query sequences, length of transmembranes, length of cytoplasmic and non-cytoplasmic regions with corresponding sequences. The result can also be downloaded from the server in FASTA format or can be directly uploaded to another server(s). TM-Aligner can be accessed through http://lms.snu.edu.in/TM-Aligner/.
